# HBV status modulates transaminase decrease after switching from tenofovir disoproxil fumarate to tenofovir alafenamide in people with HIV

**DOI:** 10.1093/jac/dkag045

**Published:** 2026-02-10

**Authors:** Giuseppe Lapadula, Alessandro Soria, Laura Antolini, Alban Rugova, Elisa Colella, Francesca Sabbatini, Nicola Squillace, Luca Mezzadri, Silvia Limonta, Anna Cappelletti, Alice Ranzani, Paolo Bonfanti

**Affiliations:** School of Medicine, University of Milano-Bicocca, Milan, Italy; Infectious Diseases Unit, IRCCS Fondazione San Gerardo dei Tintori, Monza (MB), Italy; Infectious Diseases Unit, IRCCS Fondazione San Gerardo dei Tintori, Monza (MB), Italy; Bicocca Bioinformatics Biostatistics and Bioimaging Center - B4, University of Milano-Bicocca, Milan, Italy; Infectious Diseases Unit, IRCCS Fondazione San Gerardo dei Tintori, Monza (MB), Italy; Infectious Diseases Unit, IRCCS Fondazione San Gerardo dei Tintori, Monza (MB), Italy; Infectious Diseases Unit, IRCCS Fondazione San Gerardo dei Tintori, Monza (MB), Italy; Infectious Diseases Unit, IRCCS Fondazione San Gerardo dei Tintori, Monza (MB), Italy; School of Medicine, University of Milano-Bicocca, Milan, Italy; Infectious Diseases Unit, IRCCS Fondazione San Gerardo dei Tintori, Monza (MB), Italy; Infectious Diseases Unit, IRCCS Fondazione San Gerardo dei Tintori, Monza (MB), Italy; Infectious Diseases Unit, IRCCS Fondazione San Gerardo dei Tintori, Monza (MB), Italy; School of Medicine, University of Milano-Bicocca, Milan, Italy; Infectious Diseases Unit, IRCCS Fondazione San Gerardo dei Tintori, Monza (MB), Italy

## Abstract

**Background:**

Switching from tenofovir disoproxil fumarate (TDF) to tenofovir alafenamide (TAF) has been associated with reduced transaminase level among people with HIV (PWH), with and without HBV. It is unclear whether the effect is mediated by HBV serostatus.

**Methods:**

We conducted a longitudinal observational study of PWH who switched from TDF to TAF between 2016 and 2023. A mixed-effects model with random intercepts assessed the effect of the switch on transaminases, including an interaction term for HBV status: chronic hepatitis B (HBsAg+), possible occult HBV infection (pOBI; HBsAg−/HBcAb+) and no HBV.

**Results:**

Among 727 individuals, 7% had chronic hepatitis B and 35% had pOBI. Switching to TAF was associated with a significant ALT decrease (*β* −3.5 UI/mL, 95%CI: −5.2 to −1.9). Compared to HBV-negative individuals, individuals with chronic hepatitis B experienced steeper ALT reduction (*β* −7.5, 95%CI: −11.8 to −3.2), while pOBI was associated with a non-significant reduction (*β* −1.9; 95%CI: −4.4–0.6; *P* = 0.133). These findings persisted after adjusting for other predictors of transaminase levels. TAF was also associated with accelerated weight gain (+0.75 kg/year, 95% CI: 0.63–0.87) and a transient drop in the Hepatic Steatosis Index (−0.22; 95% CI: −0.38 to −0.06) followed by an annual increase thereafter (+0.18/year; 95% CI: 0.10–0.27).

**Conclusions:**

Switching from TDF to TAF is associated with modest but statistically significant ALT reduction in PWH, especially in HBsAg + individuals. Our findings suggest that TAF may represent a favourable option in this subgroup, although potential metabolic consequences warrant close monitoring.

## Introduction

Tenofovir alafenamide (TAF), a prodrug of tenofovir, represents a pharmacologically enhanced alternative to tenofovir disoproxil fumarate (TDF) for treating HIV and HBV. It was specifically developed to minimize renal and bone toxicity associated with TDF, since its formulation demonstrates greater intracellular stability, enabling administration at lower doses with reduced systemic exposure. These pharmacokinetic improvements are linked to a potentially more favourable bone and renal safety profile, with studies reporting reduced markers of bone metabolism and renal dysfunction.^1–3^

Clinical data in persons with HBV monoinfection indicate unexpected reductions in transaminase levels following the switch from TDF to TAF, even among those with normal baseline values.^4–6^ Studies in persons with HIV (PWH) have shown similar trends, with significant decreases in ALT and AST levels post-switch.^7–9^ However, conflicting findings exist, where no significant differences in transaminase trends were observed between TDF and TAF groups after adjusting for comorbidities and treatments.^10,11^ While not fully specific for liver injury, transaminases are widely used, readily available markers of hepatic inflammation and are central to monitoring liver-related complications during antiretroviral therapy.^12^

The mechanisms underlying these transaminase changes remain unclear. One hypothesis suggests that TAF’s enhanced intracellular concentrations may lead to better suppression of HBV replication, indirectly improving liver function. Alternatively, a differing effect of TDF compared to TAF in its ability to induce hepatocyte inflammation cannot be ruled out. While cumulative TDF use among PWH has been linked to a higher risk of hepatic events in PWH, independent of HBV co-infection,^13^ there is no clear evidence that TDF leads to greater liver inflammation than TAF, either through direct toxicity on hepatocytes or by inducing metabolic changes such as liver steatosis.

To better understand these dynamics and determine whether the effect of switching from TDF to TAF is mediated by HBV (either chronic overt or occult infection), we conducted an observational study on a large cohort of PWH with varying HBV statuses who switched from TDF to TAF in a real-world setting.

## Patients and methods

We conducted a longitudinal study on participants enrolled in the San Gerardo Hospital (HSG) HIV Cohort Study. The cohort includes PWH receiving care at San Gerardo Hospital, a tertiary referral centre for HIV infection. All participants provide written informed consent upon enrolment in the cohort. They are followed according to routine clinical practice, with clinical, laboratory, and treatment-related data—including new diagnoses and administered therapies—prospectively recorded in an electronic medical record system.^14^ Individuals who switched from TDF to TAF between 2016 and 2023 while their HIV-RNA was undetectable were selected and categorized based on their Hepatitis B surface antigen (HBsAg) and Hepatitis B core antibody (HBcAb) status prior to the switch. They were classified as having chronic HBV (CHBV) if they were HBsAg-positive, as having possible occult HBV infection (pOBI) if they were HBsAg-negative but HBcAb-positive, and as having no evidence of current or past exposure to HBV if they were negative for both markers. To account for ∼5% missing data on HBV status, we used multiple imputation by chained equations to generate 20 complete datasets, each containing imputed values for the missing covariate. The imputation model included the following variables as predictors: age, sex, nationality, risk factor for HIV acquisition, CD4+ count, alanine aminotransferase (ALT) and aspartate aminotransferase (AST) levels at the time of the switch, HBsAg status (positive, negative, unknown), diabetes, use of integrase strand transfer inhibitors, protease inhibitors, or non-nucleoside reverse transcriptase inhibitors. Results were pooled for Rubin’s rules and presented as percentages with 95% confidence intervals. Of note, complete-case analyses—obtained by excluding the few individuals with incomplete HBV status data—were also conducted, yielding results entirely consistent with those obtained using multiple imputation, with very similar estimates (data not shown).

To evaluate the impact of the switch on longitudinal transaminase levels (ALT and AST levels), we used a mixed-effects model with random intercepts at the patient level, including time as a covariate. Time was measured in years, with the switch date coded as 0, negative values indicating time points prior to the switch, and positive values indicating time points after the switch. A time-dependent, non-reversible binary variable indicating the occurrence of the treatment switch was also included in the model to evaluate whether the switch exerted an immediate, fixed effect on transaminase levels. An interaction term between the switch and HBV status was included to assess whether the effect of switching varied among the three HBV groups. Furthermore, we performed multivariable models to account for patient- and treatment-related factors that could potentially influence transaminase levels. The covariates explored in the models were age, sex, HIV acquisition risk factor, nationality (Italian versus foreign-born), diabetes status, the specific antiretroviral treatment regimen administered alongside TAF/TDF, CD4+ T-cell count, BMI and the calendar year at the time of the switch.

Moreover, to evaluate the longitudinal effect of the therapeutic switch on body weight and BMI, we fitted a linear mixed-effects model with separate time slopes before and after the switch, excluding any fixed effect for the switch itself. This approach assumes no abrupt changes at the time of the switch, but allows for a potential change in the trajectory of weight or BMI over time following the intervention. In addition to a random intercept, we included a random slope for time at the patient level, to account for the considerable inter-individual variability in weight change trajectories following TAF introduction, observed in clinical practice.^15^ Random effects were modelled using an unstructured covariance matrix, allowing for correlation between individual intercepts and slopes.

Finally, considering the potential unfavourable metabolic effects associated with the switch to TAF, particularly its link to weight gain, we sought to evaluate whether the beneficial effects of TAF on liver function, as reflected in transaminase levels, could be counterbalanced by negative metabolic consequences on hepatic steatosis. To address this, we used the Hepatic Steatosis Index (HSI) as a surrogate marker of liver steatosis risk. A linear mixed-effects model with random intercept and random slope at the patient level was applied to assess changes in HSI over time, incorporating an interaction between switch status and time to allow for differential trends after the switch, while also accommodating a potential immediate change at the time of treatment modification, due to the effect on transaminase levels. The analysis was restricted to the subset of individuals with available BMI data, as BMI is a key component of the HSI formula. The HSI was calculated as follows: HSI = 8 × (AST/ALT) + BMI + 2 (if female) + 2 (if diabetes mellitus).^16^

All analyses were conducted using Stata/MP 17.0 Parallel Edition (StataCorp LLC, College Station, TX, USA).

## Results

A total of 727 individuals from the HSG cohort had switched treatment from TDF to TAF between 2016 and 2023 and were therefore included in the study. Most of them were male (74%), with a mean age of 49 years. The median CD4+ T-cell count at the time of the switch was 713 cells/mm^3^ and transaminase levels before the switch were within normal limits for the vast majority, with a median value of 22 IU/mL for ALT and 21 IU/mL for AST. HBV status classification was unavailable for 38 individuals (5%), including 24 with negative HBsAg but missing HBcAb and 14 with both markers missing. After multiple imputation, 7% were classified as having CHB, 35% as pOBI and 58% showed no serological evidence of past HBV infection. These and other baseline characteristics are presented in Table 1.

**Table 1.** Characteristics of patients at the time of the switch from TDF to TAF

Patients’ characteristics (*n* = 727)	
Age, years—median (IQR)	49 (41–55)
Sex, male—*n* (%)	540 (74.3%)
Risk factor for HIV acquisition—*n* (%)	
MSM	196 (27%)
Heterosexual intercourses	322 (44.3%)
Injecting drug use	90 (12.4%)
Other/unknown	119 (16.4%)
Place of birth, foreign born—*n* (%)	139 (19.1%)
Antiretroviral drugs in the regimen—*n* (%)	
Non-nucleoside reverse-transcriptase inhibitor	292 (40.2%)
Protease inhibitor	131 (18%)
Integrase-strand inhibitor	310 (42.6%)
Ritonavir or cobicistat as *booster*	257 (35.3%)
HBV serostatus (observed data)—*n* (%)	
Chronic hepatitis B (HBsAg+)	51 (7%)
Possible occult hepatitis B (HBsAg−/HBcAb+)	238 (32.7%)
No hepatitis B exposure (HBsAg−/HBcAb−)	400 (55%)
Missing^a^	38 (5.3%)
HBV serostatus (estimated)^b^—% (95%CI)	
Chronic hepatitis B (HBsAg+)	7% (5.1–8.9)
Possible occult hepatitis B (HBsAg−/HBcAb+)	34.7% (30.1–38.6)
No hepatitis B exposure (HBsAg−/HBcAb−)	58.3% (54.3–62.2)
Diabetes—*n* (%)	104 (14.3%)
Year of the switch from TDF to TAF—*n* (%)	
2016–2017	392 (53.9%)
2018–2019	237 (32.6%)
2020–2023	98 (13.5%)
BMI^c^, kg/m^2^—median (IQR)	24.5 (22.2–27.3)
CD4+ T-cell count, cells/mm^3^—median (IQR)	713 (517–910)
ALT level, IU/mL—median (IQR)	22 (16–30)
AST level, IU/mL—median (IQR)	21 (18–27)

ALT, alanine transferase; AST, aspartate amino transferase; BMI, body mass index; CI, confidence interval; HBcAb, hepatitis B core antibodies; HBsAg, hepatitis B surface antigen; HBV, hepatitis B virus; MSM, men who have sex with men; TAF, tenofovir alafenamide; TDF, tenofovir disoproxil fumarate; IQR, interquartile range.

^a^HBV status was missing in 24 cases with negative HBsAg and missing HBcAb, and in 14 cases with both HBsAg and HBcAb missing.

^b^Results from the Multiple Imputation by Chained Equations (MICE) model across 20 datasets were pooled using Rubin’s rules and presented as percentages with 95% confidence intervals.

^c^BMI data available for 661 individuals.

Using a mixed model with random intercepts at the patient level, we observed a significant decrease in ALT levels following the switch to TAF (*β* −3.5 U/L, 95%CI: −5.2 to −1.9, *P* < 0.001). ALT levels also showed a gradual decline over time, with a mean reduction of −0.9 U/L per year (*P* < 0.001), independent of the treatment status. However, there was no evidence that the rate of ALT decline differed before and after the switch (interaction term: *P* = 0.229).

To investigate the potential modifying effect of HBV serostatus, we fitted an additional model including an interaction term between HBV serostatus and the switch (full results in Table 2). This analysis revealed a significant interaction, indicating that compared with individuals without HBV, those with CHB experienced an additional, significantly steeper ALT decrease upon switch (*β* −7.5, 95% CI: −11.8 to −3.2; *P* = 0.001), whereas pOBI showed a smaller, non-significant reduction (*β* −1.9; 95% CI: −4.4 to 0.6; *P* = 0.133). In absolute terms, switching from TDF to TAF was associated with a mean ALT reduction of 2.1 U/L (95% CI: −4 to −0.1) among HBV-negative individuals, 4 U/L (95%CI: −6.2 to −1.7) among those with pOBI and 9.6 U/L (95%CI: −13.7 to −5.4) among those with CHB, the latter being significantly greater than the reduction observed in HBV-negative individuals. Correspondingly, mean ALT levels decreased from 30 U/L (95%CI: 27–32) to 27 U/L (95%CI: 25–30) among HBV-negative individuals, from 32 U/L (29–35) to 28 U/L (25–31) among those with pOBI and from 36 U/L (30–42) to 26 U/L (20–32) among those with CHB.

**Table 2.** Mixed-effects regression analysis predicting ALT levels

Covariate	Coefficient	95%CI	*P*
Switch (TDF to TAF)	−2.1	−4 to −0.1	0.038
HBV serostatus (versus HBV negative)			
pOBI	2.6	−0.9 to 6	0.149
Chronic hepatitis B	6.4	−0.3 to 12.8	0.051
Interaction: Treatment Switch × HBV serostatus			
Switch × pOBI	−1.9	−4.4 to 0.6	0.133
Switch × Chronic hepatitis B	−7.5	−11.8 to −3.2	0.001
Time (per year)	−0.7	−1 to −0.4	<0.001
Constant	29.6	27.4–31.8	<0.001

Random effects: The standard deviation of the random intercepts at the patient level was 19.1 (95% CI: 18–20.3) and the residual standard deviation was 32.3 (95% CI: 31.9–32.6).

HBV, hepatitis B virus; pOBI, possible occult hepatitis B virus infection; TAF, tenofovir alafenamide fumarate; TDF, tenofovir disoproxil fumarate.

**Figure 1.**
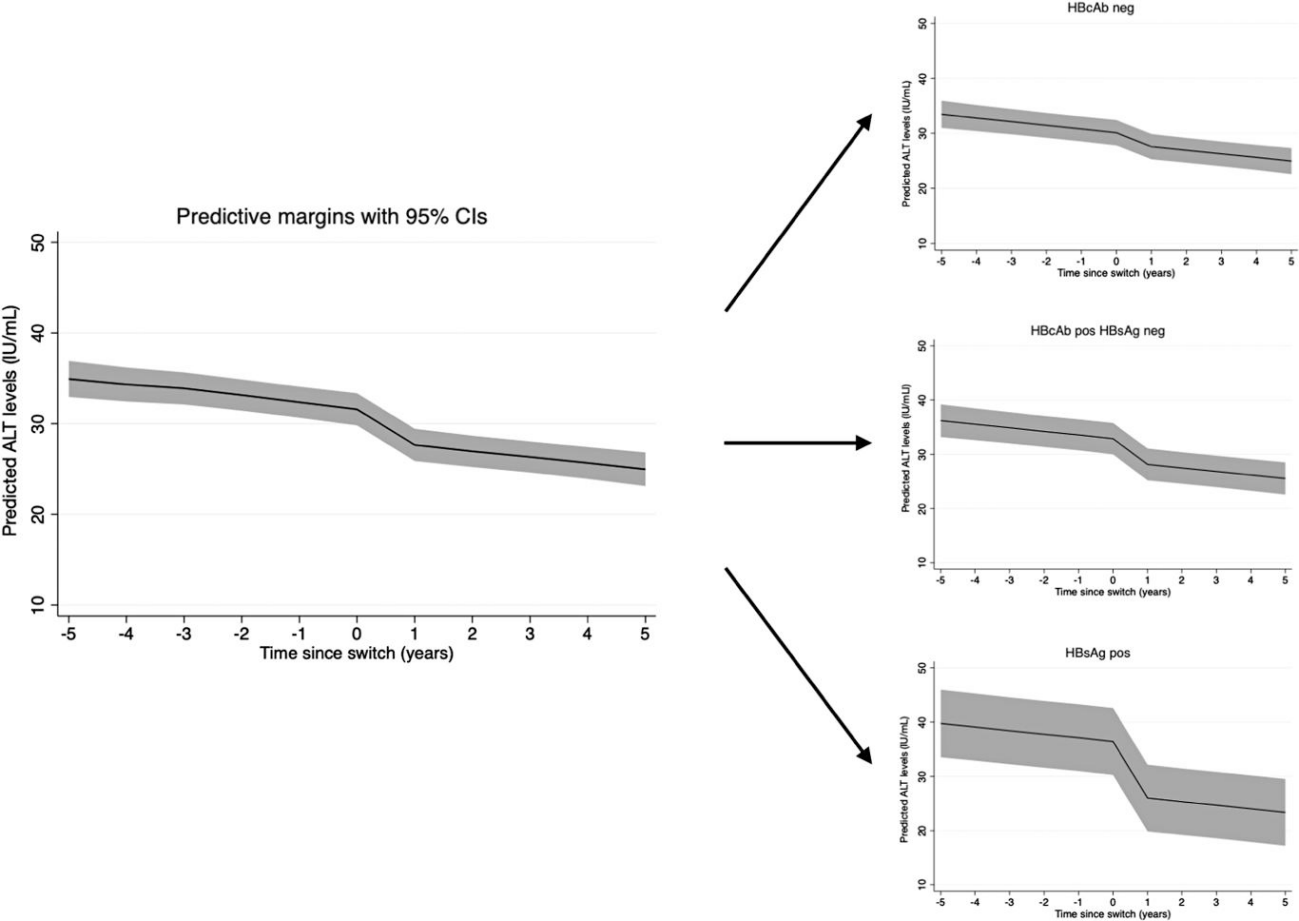
Predicted ALT values, based on marginal effects from mixed models. ALT, alanine amino transferase; CI, confidence intervals, HBcAb, hepatitis B core antibodies; HBsAg, hepatitis B surface antigen; neg, negative; pos, positive.

Figure 1 shows the estimated ALT trajectories, overall and by HBV serostatus, based on marginal effects from the mixed-effects model adjusted for time, switch, HBV serostatus and their interaction, as described above.

Similarly, in the mixed model for AST, we observed a significant decrease in AST levels following the switch to TAF (*β* −2.3 U/L, 95% CI: −3.6 to −1.0, *P* < 0.001). AST levels also declined gradually over time, with a mean reduction of −0.7 U/L per year (*P* < 0.001), independent of treatment status. However, in contrast to ALT, the rate of yearly AST decline differed significantly before and after the switch, as indicated by the interaction term (*β* + 0.5 U/L, 95% CI: 0.03–0.9, *P* = 0.036), suggesting a modest attenuation of AST reduction after switching. The AST model including the HBV serostatus interaction revealed a pattern similar to that observed for ALT: compared with persons without HBV, those with CHB experienced an additional, significantly steeper AST decrease after switching (*β* −4.7, 95% CI: −7.9 to −1.4, *P* = 0.005), while pOBI showed no significant reduction (*β* −0.6, 95% CI: −2.5 to 1.2, *P* = 0.515).

The association between switching from TDF to TAF and the reduction in ALT levels remained consistent after adjusting for other covariates. In individual models that included each covariate along with switch status, time and their interaction, several factors were found to be independently associated with ALT levels. Specifically, male sex was associated with higher ALT levels (*β* + 10.6, 95% CI: 7.2–14.1; *P* < 0.001), as was intravenous drug use (IVDU) as a risk factor for HIV acquisition (*β* + 4.6, 95% CI: 0–9.1; *P* = 0.050). Calendar year also showed a significant association: compared to individuals who switched before 2018, lower ALT levels were observed among those who switched in 2018–2019 (*β* −3.8, 95% CI: −7.2 to −0.5) and in those who switched from 2020 onwards (*β* −8.7, 95% CI: −13.4 to −3.9). Eventually, BMI showed a modest association, with ALT levels increasing by 0.37 U/L per unit increase in BMI (95% CI: 0.02–0.72; *P* = 0.038). Notably, in none of these models did any of the covariates significantly modify the effect of the switch or the switch-by-time interaction. In a final multivariable model adjusting for sex, age, IVDU status, calendar year, BMI and type of antiretroviral therapy, the effect of switching from TDF to TAF on ALT levels remained significant and robust (*β* −3.6, 95% CI: −5.3 to −1.9; *P* < 0.001). Finally, when we included the interaction term to evaluate whether HBV status modifies the effect of switching on ALT levels, the results were consistent with those of the primary model, confirming that HBV status acts as a treatment modifier even when additional covariates were taken into account. Table 3 shows the full results of this latest model. Similar results were obtained for the AST models (data not shown).

**Table 3.** Multivariable mixed-effects regression analysis predicting ALT levels

Covariate	Coefficient	95%CI	*P*
Switch (TDF to TAF)	−2.1	−4.1 to −0.2	0.034
HBV serostatus (versus HBV negative)			
pOBI	1.6	−2.2 to 5.3	0.425
Chronic hepatitis B	5.7	−0.6 to 12.1	0.076
Interaction: Treatment Switch × HBV serostatus			
Switch × pOBI	−1.8	−4.3 to 0.7	0.165
Switch × Chronic hepatitis B	−7.5	−11.8 to −3.2	0.001
Time (per year)	−0.7	−1 to −0.4	<0.001
Male gender	10.1	6.6–13.6	<0.001
Age (per 10 years increase)	−1.6	−3 to −0.1	0.030
IVDU as HIV risk factor	3.1	−1.8 to 8	0.215
Calendar year at switch (versus 2016–2017)			
2018–2019	−2	−5.6 to 1.5	0.261
2020–2023	−7.1	−11.8 to −2.4	0.003
ARV regimen (versus INSTI)			
NNRTI	−3.7	−7.3 to −0.1	0.041
PI	−0.5	−4.8 to 3.8	0.817
Constant	33.1	25.6–40.6	<0.001

Random effects: The standard deviation of the random intercepts at the patient level was 18.5 (95% CI: 17.3–19.6) and the residual standard deviation was 32.4 (95% CI: 32–32.8).

ARV, antiretroviral; HBV, hepatitis B virus; INSTI, integrase strand transfer inhibitors; IVDU, intravenous drug users; NNRTI, non-nucleoside reverse transcriptase inhibitors; PI, protease inhibitors; pOBI, possible occult hepatitis B virus infection; TAF, tenofovir alafenamide fumarate; TDF, tenofovir disoproxil fumarate.

Regarding weight gain, patients experienced a statistically significant but modest increase in body weight prior to the switch, with an average annual gain of +0.38 kg/year (95% CI: 0.24–0.51). Following the switch to TAF, the rate of weight gain significantly accelerated to +0.75 kg/year (95% CI: 0.63–0.87). These changes in weight corresponded to an average annual increase in BMI of 0.12 kg/m^2^/year (95% CI: 0.08–0.16) before the switch, which rose to 0.26 kg/m^2^/year (95% CI: 0.22–0.3) after the switch.

To explore the trajectory of hepatic steatosis risk, by means of HSI, and assess whether the rate of change differed before and after the therapeutic switch, we fitted a linear mixed-effects model, allowing for random intercepts and random slopes at the patient level and including an interaction term between switch status and time. The model was estimated using observations from 643 individuals with available BMI measurements. The results indicated that the switch to TAF was associated with a statistically significant reduction in HSI (*β* −0.22; 95% CI: −0.38 to −0.06; *P* = 0.007). However, this was followed by a steeper annual increase thereafter, as shown by a statistically significant interaction between treatment period and time (*β* + 0.18; 95% CI: 0.10–0.27; *P* < 0.001). These findings suggest a complex dynamic, in which the switch was initially associated with a modest decline in HSI, possibly reflecting a better AST-to-ALT ratio, but was subsequently followed by a progressive increase in hepatic steatosis risk over time, potentially driven by weight gain.

## Discussion

Tenofovir disoproxil-fumarate remains the most widely used formulation of tenofovir globally, despite the availability of TAF-based regimens. The theoretical advantages of TAF over TDF in terms of renal and bone safety are well established, with TAF demonstrating reduced renal and bone toxicity due to its pharmacokinetic profile, which allows for lower doses while maintaining effective intracellular concentrations.^2,3,17^ However, these benefits are counterbalanced by metabolic concerns, particularly the potential for weight gain, which could, over time, increase the risk of metabolic complications, such as diabetes or hepatic steatosis.^15,18^ The shift from TDF to TAF, therefore, has been largely driven by the availability of co-formulations, as opposed to clear guidelines on when such a switch should be made. The lack of definitive recommendations underscores the advantage of identifying categories of individuals that would benefit from more specific clinical criteria for switching.

In this study, we explored the effects of switching from TDF to TAF on liver function in persons with HIV, with or without concurrent HBV infection. Our findings suggest that the switch to TAF was associated with a reduction in liver inflammation, as indicated by statistically significant decreases in transaminase levels (ALT and AST). This improvement was particularly pronounced in individuals with chronic HBV, while individuals without evidence of HBV co-infection showed much less improvement. Notably, individuals with possible occult HBV demonstrated a trend towards a further reduction in transaminase levels compared to those without HBV, although this difference did not reach statistical significance. These observations are consistent with prior studies, which have shown that TAF leads to a more favourable biochemical profile among persons with HBV mono-infection.^4–6^ Observational studies have shown similar trends also in persons living with HIV, although these studies did not specifically explore whether the observed effects were mediated by HBV status.^7–9^ In a recent randomized controlled trial comparing the effects of TAF and TDF on individuals with HIV and HBV coinfection, individuals receiving TAF had higher rates of HBsAg seroconversion and a higher rate of ALT normalization compared to those receiving TDF.^19^

Taken together, these findings suggest that the improvement in transaminase levels observed following the switch from TDF to TAF in PWH is, at least in part, modulated by the presence of HBV. Specifically, TAF appears to be associated with a better hepatic safety profile and lower levels of liver inflammation in individuals with chronic hepatitis B. This effect may reflect, at least in part, an enhanced antiviral activity against HBV, potentially involving mechanisms that are not fully captured by plasma HBVDNA measurements. The observed differences between TAF and TDF could be related to the higher intracellular concentrations of TAF in hepatocytes, which may confer greater ability to suppress HBV replication. Indeed, Murakami *et al.* demonstrated that TAF results in higher and more persistent levels of the active metabolite tenofovir diphosphate (TFV-DP) in the liver compared to TDF.^20^ This is due to TAF's greater intracellular stability and efficient hepatic delivery, which allows for lower dosing and reduced systemic exposure while achieving higher intrahepatic concentrations. Podany *et al.* also found that after switching from TDF to TAF, cell-associated TFV-DP concentrations significantly increased, suggesting better intracellular drug delivery and greater antiviral potency.^21^

Given these findings, switching to TAF may offer advantages for individuals with HIV/HBV coinfection, and its use should be carefully considered in clinical practice. However, while TAF appears to reduce liver inflammation among individuals with overt chronic hepatitis B, its effects in those with possible occult HBV infection warrant further investigation. Our study showed a trend towards a greater reduction in transaminase levels in the pOBI group compared to HBV-negative individuals, although this difference did not reach statistical significance. This effect may also be related to the improved antiviral efficacy of TAF against HBV in these individuals. However, our findings are not definitive and require confirmation in larger cohorts, particularly among individuals with clearly defined occult HBV, using high-sensitivity HBV DNA testing methods. The current definition of pOBI based solely on HBcAb status is inherently imprecise, as only a subset of individuals with prior HBV exposure may have true occult HBV at the hepatocyte level, detectable only through more sensitive techniques such as the detection of replication-competent cccDNA in hepatocytes. Therefore, our results should be interpreted with caution to avoid overestimating the clinical relevance of this finding.

It should be noted that a reduction in transaminase levels after the switch was observed across the entire study population, even after adjusting for HBV status. Therefore, a direct effect of TDF compared to TAF in inducing liver inflammation cannot be excluded. It is worth noting that a previous observational study has indeed associated cumulative TDF use with end-stage liver disease in persons with HIV, independent of HBV co-infection.^13^ The exact mechanisms by which TDF could be associated with increased liver events are not fully elucidated. It is hypothesized that TDF may contribute to liver toxicity through mechanisms such as mitochondrial toxicity, which can lead to hepatocyte injury and subsequent liver disease progression. Additionally, TDF has been associated with renal toxicity, which can indirectly affect liver function and exacerbate liver disease. In any case, our findings are consistent with the hypothesis that switching from TDF to TAF could reduce hepatocellular injury also among individuals without active HBV replication, although further research is needed to confirm this effect in larger cohorts.

Our study confirmed previous evidence reporting a significant increase in weight following the switch to TAF.^15,18,22–25^ Unfortunately, weight gain can be associated with a broader metabolic deterioration, particularly if it leads to overweight or obesity. This, in turn, may contribute to the development of harmful metabolic complications for the liver, such as diabetes, insulin resistance and hepatic steatosis, which can further exacerbate liver damage. Consistently, when we explored the effect of the switch from TDF to TAF on HSI, as a surrogate marker of liver steatosis risk, we found a transient favourable impact of the switch on the index, followed by a subsequent increase over time. This pattern supports the hypothesis that the initial benefits of TAF in improving liver function tests may be counterbalanced by long-term metabolic deterioration, particularly if weight gain leads to a higher risk of steatosis. While the use of HSI as a surrogate marker for hepatic steatosis is a limitation, as it cannot definitively diagnose metabolic dysfunction–associated steatotic liver disease and it does not directly measure fat accumulation in the liver nor is it linearly correlated with it, the results of the analysis are suggestive. The increase in BMI following the switch, which may lead to overweight or obesity, warrants close monitoring, especially as these conditions could offset the benefits of reduced transaminases.

There are other limitations of our study that merit to be acknowledged. First, its observational design, the absence of uniform criteria for switching from TDF to TAF, and the lack of a control group may have introduced biases difficult to fully account for. Nonetheless, the consistency of transaminase trajectories over the observation period, and the observed decrease following the switch, are in line with previous findings, which we further extend by suggesting a modulation according to HBV serostatus. This congruence with existing literature, together with the biological plausibility of the proposed mechanism, provides reassurance and reduces concerns about potential sources of systematic error. It is important to note, however, that the absolute reductions in ALT and AST observed in our study were, on average, modest (2–10 U/L) and often remained within the normal range. While statistically significant, the clinical relevance of these changes is uncertain, and interpretations should be made with appropriate caution.

Second, alcohol intake, along with other key determinants of transaminase trajectories—such as dietary factors and metabolic comorbidities beyond diabetes—was not systematically assessed. While these factors could influence transaminase levels, the design of the study, based on within-subject changes before and after switching from TDF to TAF, provides a degree of internal control. Since it is highly unlikely that alcohol use, dietary habits or metabolic comorbidities changed systematically in relation to the switch to TAF, we do not believe this omission biases the observed association. In addition, although differences in these factors by HBV serostatus cannot be excluded, there is no clear biological plausibility that such differences would mediate or confound the effect of HBV serostatus on the association between the switch to TAF and transaminase trajectories. As such, while this limitation may reduce the precision of our estimates, it is unlikely to have altered their validity.

In conclusion, our study reinforces the potential advantages of switching to TAF in individuals with HIV/HBV coinfection, suggesting that TAF could be a preferred option at least for those who are HBsAg-positive. This is particularly evident in terms of liver inflammation reduction and enhanced HBV suppression. However, the metabolic consequences of weight gain and the associated risk of hepatic steatosis need to be carefully monitored.

## Data Availability

The dataset generated and analyzed during the current study is not publicly available due to ethical and privacy restrictions related to hum an participant data, but de-identified data are available from the corresponding author upon reasonable request and subject to institutional and ethical approval.
